# The phylogenetic position and analysis of *Renicola* and *Apharyngostrigea* species isolated from Cattle Egret (*Bubulcus ibis*)

**DOI:** 10.1038/s41598-023-43479-y

**Published:** 2023-09-27

**Authors:** Mai A. Salem, Olfat A. Mahdy, Mohamed Shaalan, Reem M. Ramadan

**Affiliations:** 1 Department of Parasitology, Faculty of Veterinary Medicine, Cairo University, Giza, 12211 Egypt; 2 Department of Pathology, Faculty of Veterinary Medicine, Cairo University, Giza, 12211 Egypt

**Keywords:** Diseases, Pathogenesis

## Abstract

Cattle Egret (*Bubulcus ibis)* is one of the most well-known herons in Egypt. It is called the friend of the farmer because it benefits farmers and helps them get rid of insects and worms. It acts as a reservoir for many diseases. Few researchers have discussed the significance of parasitic diseases that affect this wild bird and may lead to mortalities among the population especially the importance of vital organs such as kidneys. Therefore, this study aimed to spotlight parasitic infection-affected herons in Egypt and consider the risks to this beneficial bird. During this study, 23 *Bubulcus ibis* were captured after their death from Abou Rewash Giza Governorate, Egypt, during the period from February to September (2022). *Renicola* species (spp.) and *Apharyngostrigea* spp. are two important digenean parasites that were recovered from the kidneys, and small intestine of the heron Cattle Egret (*Bubulcus ibis)* with an infection rate of (17.2%) and (11.8%) respectively. Histopathological techniques were used to assess tissue alterations while light microscopy and molecular assays were used to assess the parasites. The parasites’ morphological and morphometrical characteristics, as well as polymerase chain reaction and sequencing assays (mitochondrial sections), were investigated for the first time in Egypt. These parasites were given in-depth illustrations and drawings. The distinctive qualities of the two species were discussed. As the first record from Egypt, the nucleotide sequences discovered in this work have been uploaded into the GenBank database (accession numbers: OR021986 and OQ955829). Microscopically, the renal blood vessels had vasculitis, necrosis, and other degenerative alterations. Further research analyzing the health of various heron spp. and environmental deterioration can help to close information gaps about the interactions between parasites, their hosts, and environmental health.

## Introduction

Herons are crucial in the biological control of agricultural pests such as insects, mollusks, earthworms, and fish, and many of them serve as intermediate hosts for helminth parasites. The cattle egret (*Bubulcus ibis*) is a worldwide heron (family Ardeidae) that lives in the tropics, subtropics, and warm-temperate zones. The cattle egret (*Bubulcus ibis*) is common in Egypt^1^. In an agricultural country like Egypt, wild birds are widely distributed due to their capacity to adapt to large climatic fluctuations. Because man is frequently in touch with water, there is a risk of infection with avian parasites^2^. The order Ciconiiformes, which includes the ardeid birds, often known as egrets and herons, typically inhabit tropical and subtropical regions in wetlands, mudflats, ponds, lakes, and water reservoirs. Herons play a significant part in the biological management of agricultural pests such as insects, mollusks, earthworms, fish, reptiles, and rodents, many of which serve as intermediate hosts for the helminth parasite.

According to^3^, domestic birds can catch diseases from wild birds. For some possible zoonotic helminth spp., some ardeids can serve as the sole hosts, and they may also be involved in the spread of digenetic trematodes from freshwater fishes^4^. The impact of parasite infection may be influenced by a variety of intrinsic and external factors. Although it is widely accepted that host characteristics such as age, sex^5^, and sexual maturity^6^ influence parasite community structures and susceptibility to parasitism, studies describing these associations are limited^7^. Other infections (viral, bacterial, or fungal), immunosuppression, and environmental changes, in addition to host susceptibility, may alter parasite number, diversity, and distribution^8–10^.

Flukes from various trematode genera, including *Renicolidae* can infect the kidneys of birds. *Renicolidae*, a family of small digeneans that inhabits the renal tubules and ureters of herons, has been described^11^. Parasitism may thus impact individual organs and alter the host's physiology and ecological behavior. Data on the presence of renicolids and their effects on the kidney in *Ardea goliath* are particularly inadequate^12,13^. Other seabird species have shown severe kidney abnormalities in studies^14^. Renicolids have five life stages: eggs, miracidium, sporocysts, cercaria, metacercaria, and adult^15^. After the primary intermediate host (marine gastropods) ingests the miracidium inside the egg, the sporocysts grow. The free larval form of the parasite, cercariae, enters the second intermediate host, marine fishes, where it transforms into metacercariae^16^.

Concerning the life cycle of *Apharyngostrigea* spp., the eggs obtained from infected herons were developed into miracidia and then entered freshwater intermediate hosts (planorbid snails) from which emerge distinctive furcocercous cercariae forming metacercariae in fish or amphibians^17^. Only a few samples from the United States have had their mitochondrial (mt) DNA sequenced^18^, whereas sequences from samples from other locations are only slowly evolving nuclear rDNA markers. Effectively, this circumstance has made it impossible to analyze the geographic distribution of *Apharyngostrigea* spp. using molecular methods. In line with^17^, two species of *Apharyngostrigea* that were sampled from a variety of intermediate and final hosts in the Americas and Africa were examined morphologically and genetically.

The aim of the current study is an updated spotlight on the existence of trematodes infecting Cattle Egret (*Bubulcus ibis)* in Giza, Egypt, with a focus on their first morpho-molecular identification in Egypt and histopathological findings.

## Material and methods

### Sample collection

During this study, 23 *Bubulcus ibis* were captured after their death from Abou Rewash Giza Governorate, Egypt during the period from February to September 2022 and brought to the laboratory and immediately dissected, Buccal cavity, trachea, intestine, and kidneys were examined carefully for any parasitic infestation. The collected trematodes were fixed in 10 ml Formalin, 50 ml Ethanol, 5 ml Glacial acetic acid, and 45 ml distilled water then cleared in lactophenol or stained with acetocarmine. The specimens are measured by using a light microscope with a divided ocular lens and all measurements are done in millimeters^13,19,20^. The identification of *Renicola* spp. was confirmed and identified according to^21^.

### Molecular analysis (DNA extraction, PCR, and sequencing)

According to^7^ and^17^, trematodes were eliminated from the kidneys, and DNA was extracted using a commercial kit (DNA Blood and Tissue kit, QIAGEN®, Valencia, CA). The primers 5′ TTTTTTGGGCATCCTGAGGTTTAT 3′ and 5′ TAAAGAAAGAACATAATGAAAATG 3′ were used to conduct the amplification of the COXI (cytochrome C oxidase, subunit I) areas for *Renicola* spp. Primer was 5′ ATTAACCCTCACTAAATTATCACGTTTAGGATCATAAG 3′ and 5′ TAATACGACTCACTATAGCATGATACACTAAATTTACTCGATC 3′ in the case of *Apharyngostrigea* spp. The PCR conditions were 94 °C for 2 min, 35 cycles of 94 °C for 30 s, 50 °C for 30 s, and 72 °C for 1 min, followed by a 10-min extension at 72 °C^22^. By electrophoresis on a 2% agarose gel in a TBE buffer containing ethidium bromide (0.5 g/mL), the PCR amplicons were identified and thereafter visible under UV light. The PCR amplicons were purified with the PCR DNA and Gel Band Purification Kit (GE Healthcare, Little Chalfont, Buckinghamshire, UK) and quantified with an Invitrogen Life Technologies Qubit® Fluorometer (Eugene, OR, USA). The original forward and reverse primers used in the initial PCR experiment have been utilized to sequence the PCR amplicons using a BigDye® Terminator Cycle Sequencing Kit in an Applied Biosystems Genetic Analyzer sequencer. The assembled sequences were examined and aligned using the BLAST tool found at (https://blast.ncbi.nlm.nih.gov/Blast.cgi) against other pertinent sequences that had been deposited in GenBank^23^. The GenBank database has received the nucleotide sequences obtained in this investigation. The GenBank database received the edited COXI sequences in order to assign accession numbers. The Neighbor-Likelihood and Maximum Likelihood methods in MEGA (version 11) were used to create the phylogenetic trees based on nucleotide sequences^24^. The neighbor-joining and Maximum Likelihood phylogenetic analysis were used to determine the evolutionary relationships between the two present sequences of *Renicola* and *Apharyngostrigea* spp. versus correlated sequences.

### Pathological examination

According to the^25^ procedure, samples were obtained from each infected kidney tissue of several birds preserved in 10% buffered formalin. Tissues were fixed in formalin for at least 24 h. Processed samples were cut with semi-automated microtome at a thickness of 5 µm. For histological investigation using light microscopy, samples were prepared using the traditional paraffin embedding technique, sectioned at 4–5 µm, and stained with hematoxylin and eosin stain. Using an Olympus CX41 microscope, the affected organs were examined and captured on camera.

### Statistical analysis

Using SPSS 27 (IBM, NY, USA), The mean, maximum, and minimum values associated with every character, as well as the length/width ratios for the suckers and the body, were determined by a statistical description^26,27^.

### Ethical approval

All experimental protocols were approved by the guidelines of the ethical committee (Institutional Animal Care and Use Committee), Faculty of Veterinary Medicine, Cairo University (Vet CU. IACUC-08072023699).

## Results

### Morphological characteristics

The present study revealed two digenetic trematodes collected from Cattle Egret (*Bubulcus ibis).* The incidence of infected birds was 17.2% and 11.8% with *Renicola* spp. and *Apharyngostrigea* spp. respectively.

**Figure 1:**
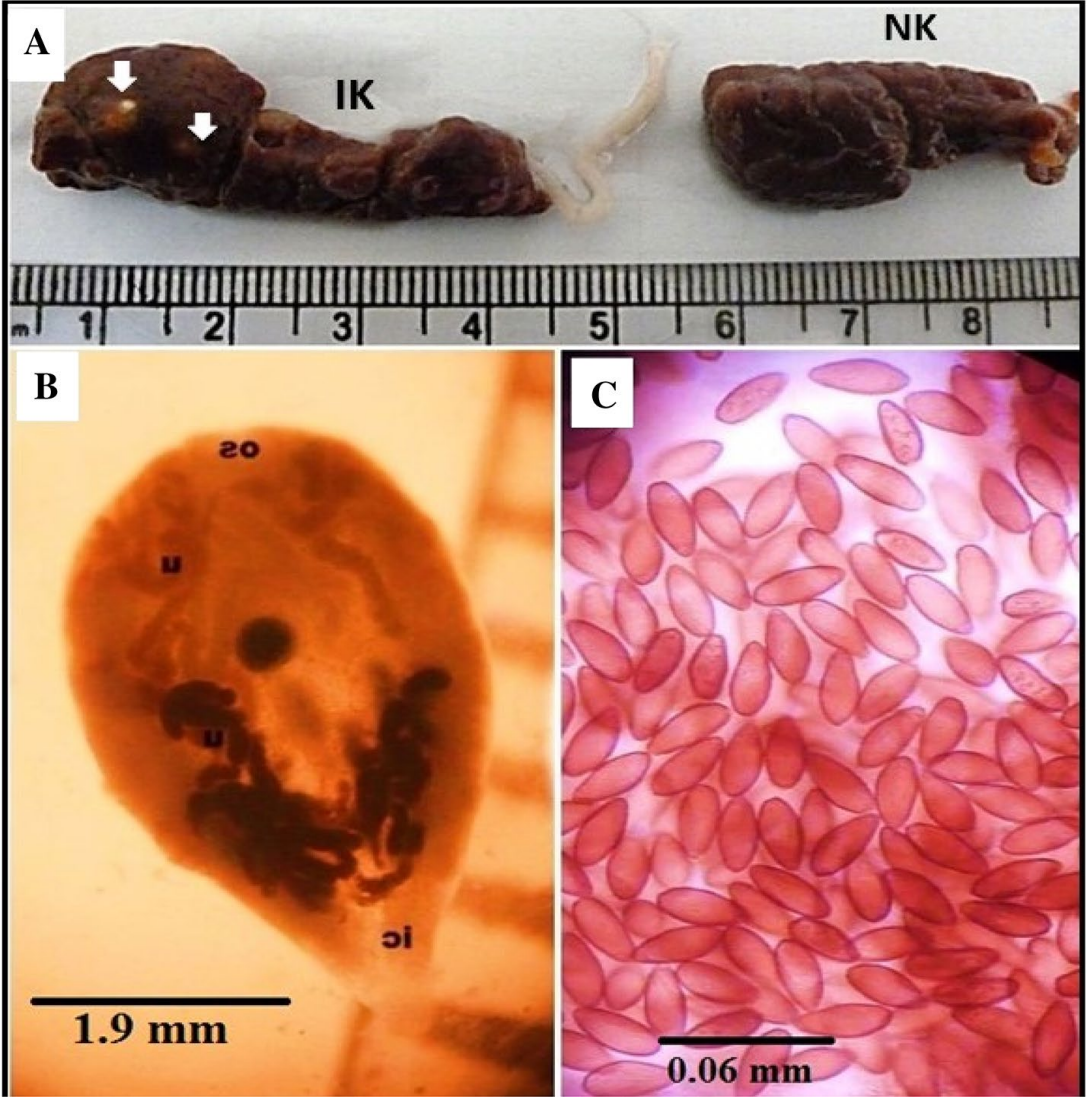
(**A**) Fresh specimens of Kidney of Cattle Egret (*Bubulcus ibis)* infected kidney (IK- arrows) with the *Renicola* parasitic cyst and normal Kidney (NK) are observed macroscopically. (**B**) Fresh specimens of *Renicola* spp. from freshly dead Cattle Egrets. (**C**) *Renicola* spp. Eggs. *IK* infected kidney; *NK* normal kidney.

**Figure 2:**
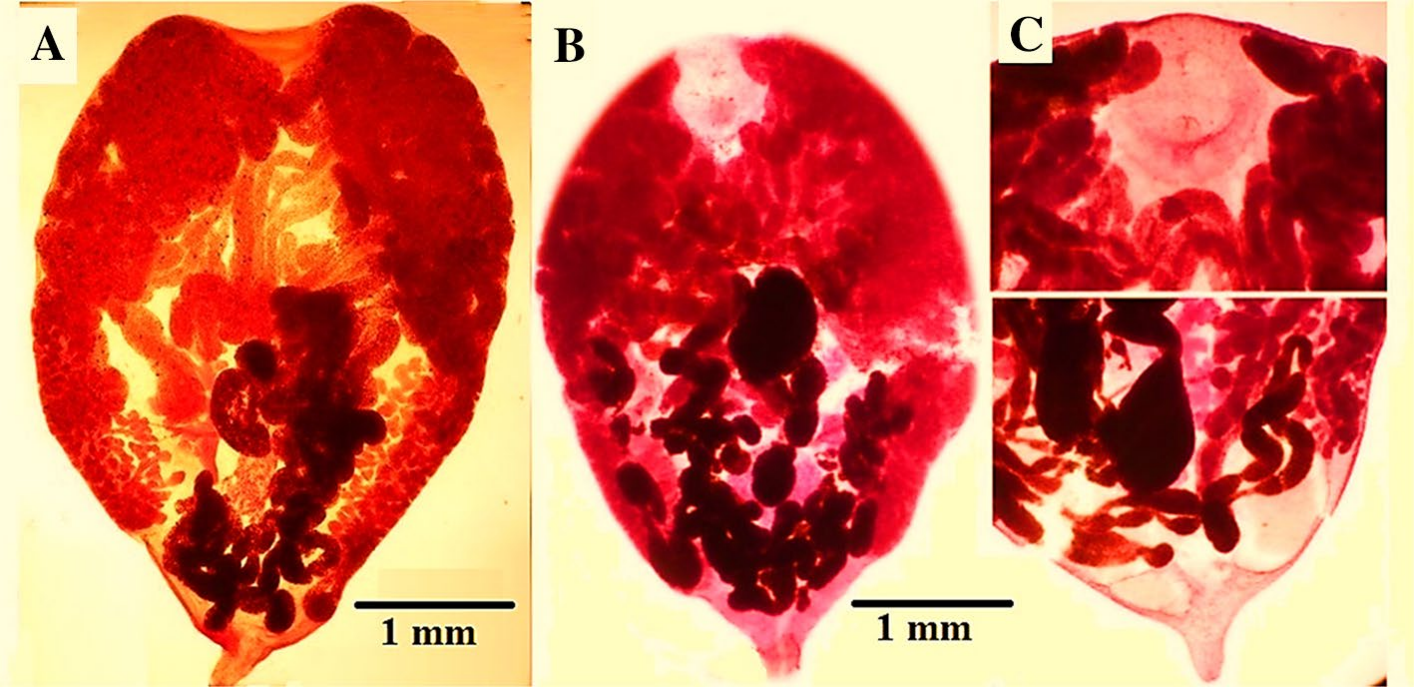
Representative microphotographs of *Renicola* spp. analyzed in this study from Cattle Egret (*Bubulcus ibis).*

**Figure 3:**
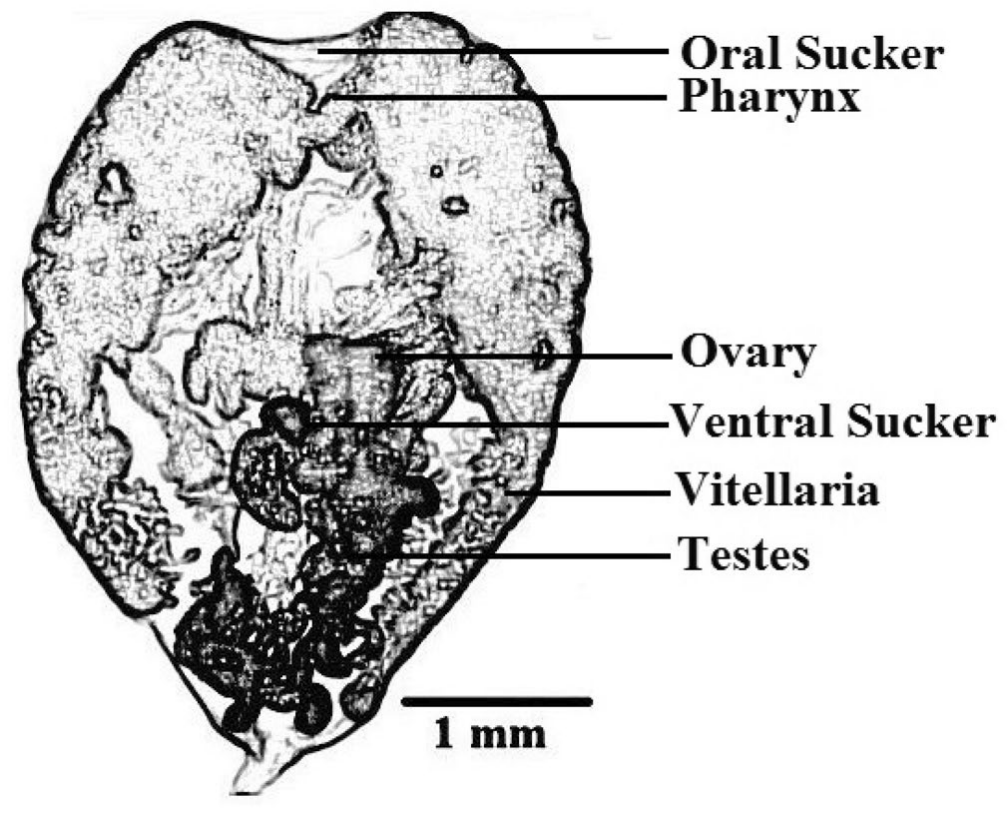
Diagrammatic drawing of *Renicola* spp.

The group of specimens (15 worms) obtained from the kidneys of the examined Cattle Egret *(Bubulcus ibis)* proved to belong to the family Renicolidae, genus *Renicola* and had the following morphological descriptions (Figs. 1, 2 and 3). The body is pyriform in shape, widest anteriorly and tapering posteriorly to produce a caudal prolongation. The length ranges from 4.0 to 5.0 (mean 4.7) mm, and the maximum breadth ranges from 3.1 to 3.5 (mean 3.2) mm. The spines cover the cuticle. The ventral sucker has a diameter of 0.1–0.2 mm. The oral sucker is big and subterminal, measuring 0.5–0.8 × 0.6–0.9 mm (mean 0.67 × 0.81) in size. The sucker's lumen is funnel-shaped and opens directly into the elongated pharynx, which measures 0.13–0.27 × 0.15–0.17 mm (mean 0.22 × 0.16). The wide caeca divides right after the pharynx and extends back to the back end of the body, almost entering the caudal extremity. The division of the intestinal caeca is disguised by the anterior coils of the uterus. The right testes are covering the ventral sucker, and the two testes look like two huge masses. The ovary is heavily branched and extends diagonally from the right testes to the left side of the body, in front and behind the ventral sucker. It seems to be made up of several distinct, large follicles. In the linear distribution, vitellaria takes the shape of coarse follicles and covers two lateral fields of the body. The uterus, which coils to take up the majority of the worm’s body, is large and developed. The mature egg has a thin, light brown, operculated shell and is tiny. It weighs 0.032 × 0.012–0.037–0.014 (mean 0.034 × 0.013) mm and has a miracidium within.

Other specimens (11 worms) were also collected from the kidneys and belonged to the same genus but with some morphometrical differences (Figs. 2, 3). The oral sucker is larger and spherical in shape, it measures 0.87–1.1 (mean 0.98) mm, in diameter. The pharynx is round in shape and present. At the level of the oral sucker. It measures 0.22–0.27 (mean 0.25) in diameter, Ventral sucker is rounded and lies between the testes, behind the vitelline duct, it measures 0.09–0.13 (mean 0.11) mm. in diameter. The two testes lie side by side in a V-shaped manner immediately behind the transverse vitelline duct. The branched ovary occupies a narrow area at the right of the midbody and crosses the transverse vitelline duct. Vitellaria is in the form of coarse follicles that occupy the middle third of the two lateral fields. The egg is ovoid, light in color, thick-shelled shelled and operculated, it contains a miracidium and measures 0.026 × 0.012–0.019 (mean 0.037 × 0.016) mm.

**Figure 4:**
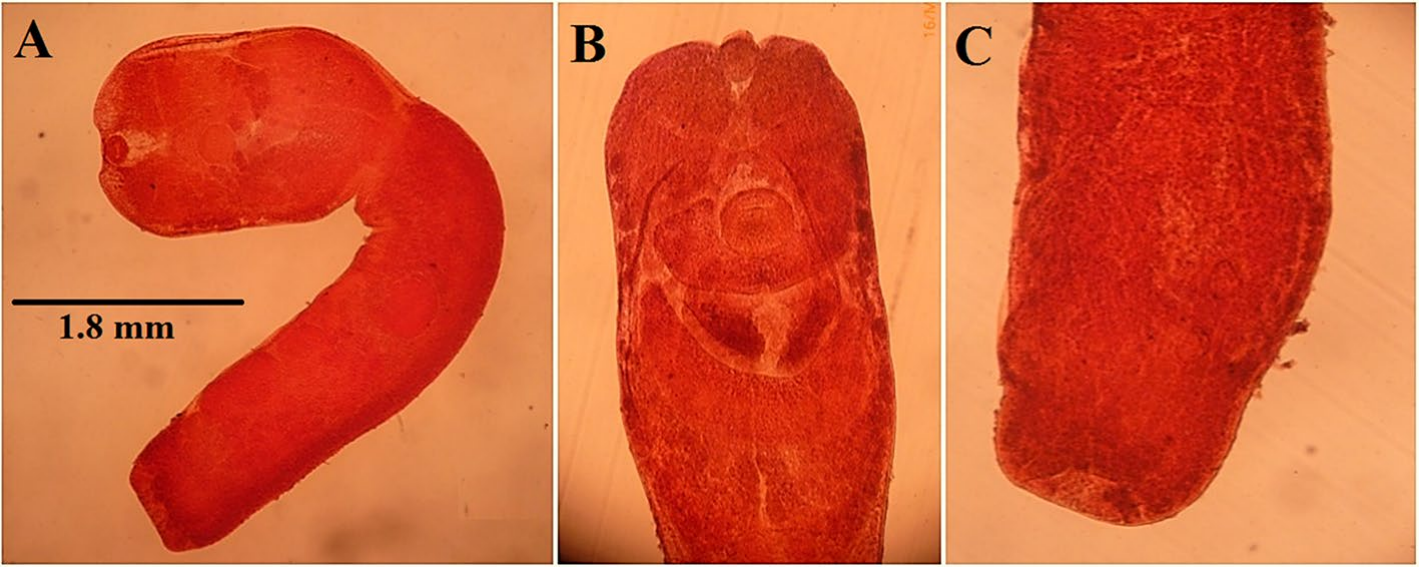
*Apharyngostrigea* spp. from Cattle Egret (*Bubulcus ibis)*, Egypt. (**A**) Whole mount. (**B**) Fore body. (**C**) Hind body.

**Figure 5:**
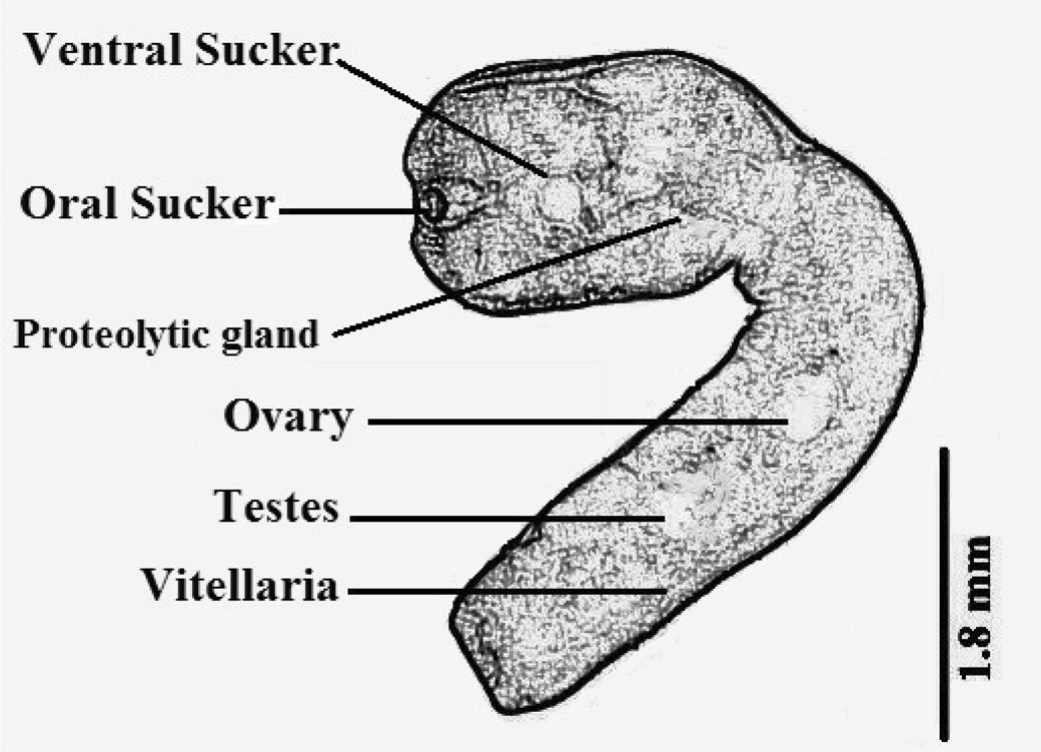
Diagrammatic drawing of *Apharyngostrigea* spp.

On the other hand, there were (10 worms) collected from the intéstine and proved to belong family Strigeidae, genus *Apharyngostrigea*. and had the following morphological characteristics (Figs. 4, 5). The body of the parasite is divided into a forebody ending at the posterior margin of the tribocytic organ and a cylindrical hind body which is larger than the forebody. The parasite is 2.2–4.2 (mean 3.6) mm, in length. The ratio of the fore/hind body is 1:2. The body is covered with minute spines. Suckers are well developed, oval in shape and ventral larger than oral measuring 0.30–0.35 × 0.28–0.38 mean (0.33 × 0.29) mm. Pharynx absent. Proteolytic gland in the intersegmental region. A small kidney-shaped ovary lies in the midline of the hind body. It occurs at a distance of 1.09 mm. from the posterior end of the tribocytic organ and measures about 0.13–0.15 × 0.24–0.32 (mean 0.135 × 0.29) mm. The testes are two multilobulated, and tandem in position. Vitellaria, are large follicles that occupy both fore and hind bodies. Eggs are oval in shape, thin shells Yellowish in color and measure 0.089–0.115 × 0.64–0.077 (mean 0.10 × 0.073) mm. It contains an ill-developed embryo.

### Molecular identification

**Figure 6:**
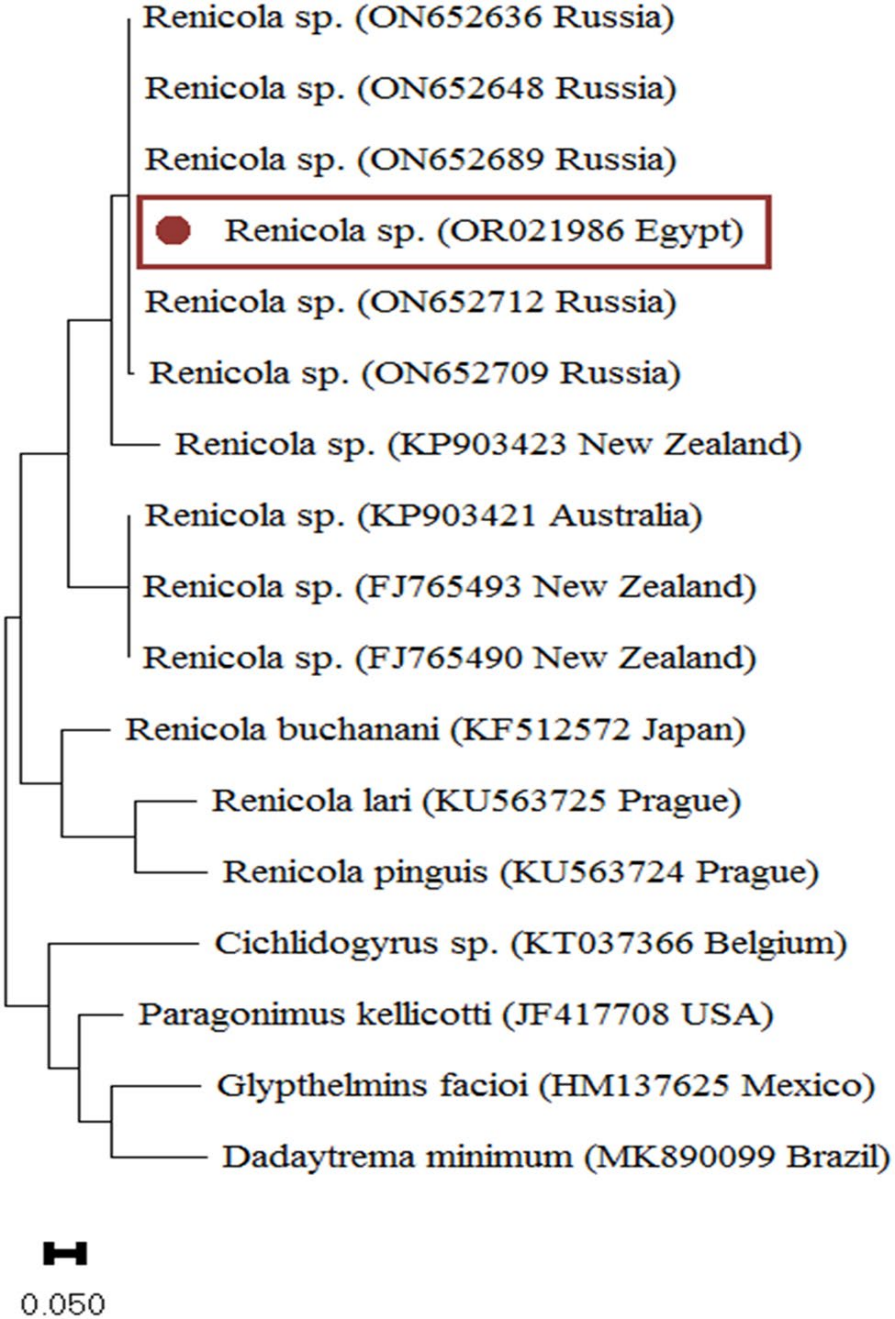
*Renicola* spp. phylogenetic analysis using COXI regions. The Maximum Likelihood method was used to build the tree. The number of nucleotide substitutions per site is shown by the scale bar at the base of the tree. The GenBank accession numbers of the strains were provided, and the sequences from this investigation are identified by a filled circle. Outgroups included the *Cichlidogyrus* spp., *Dadaytrema minimum*, *Glypthelmins facioi* and *Paragonimus kellicotti*.

**Figure 7:**
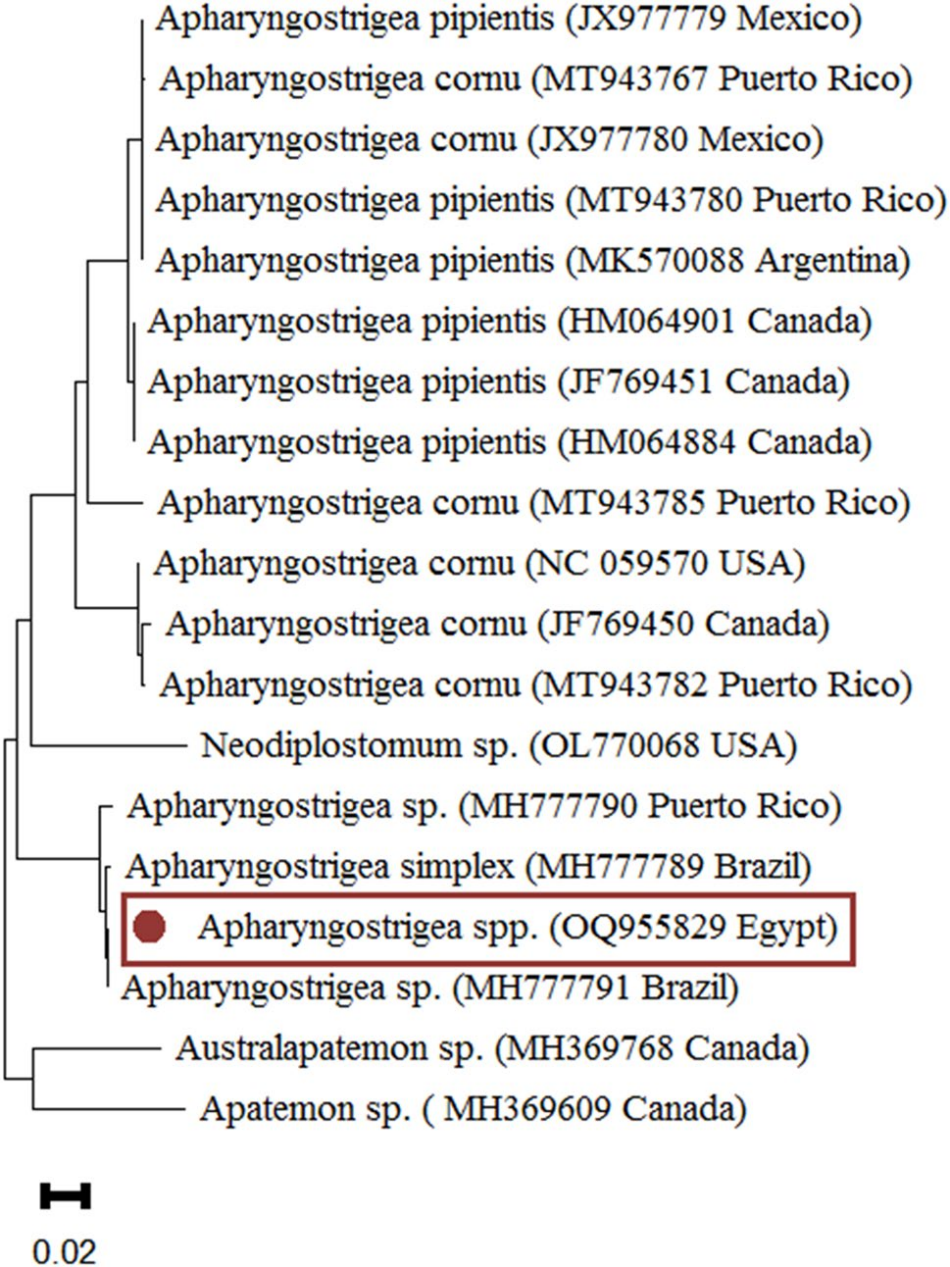
*Apharyngostrigea* spp. phylogenetic study based on COXI regions. The Neighbor-Joining method was used to build the tree. The number of nucleotide substitutions per site is shown by the scale bar at the base of the tree. The GenBank accession numbers of the strains were provided, and the sequences from this investigation are identified by a filled circle. Outgroups included the *Australapatemon* spp., *Neodiplostomum* spp. and *Apatemon* spp.

Interestingly, two main clades were identified by the phylogenetic analysis of COXI gene sequences. The amplified products of COXI had 434 bp and 428 from *Renicola* spp. and *Apharyngostrigea* spp., respectively. The sequence of COXI locus of our *Renicola* spp. (accession number: **OR021986**) showed higher nucleotide similarity 100%, with accession number: (ON652712; ON652665) and 99.57% with accession number: (ON652700; ON652648). The lower nucleotide similarity was with *R. buchanani* (87.37%; accession number: KF512572), *R. lari* (86.02%; accession numbers: KU563725) and *R. pinguis* (84.54%; accession numbers: KU563724). In the case of *Apharyngostrigea* spp., the sequence of COXI locus (accession number: **OQ955829**) showed higher nucleotide similarity 100%, with accession number: (MH777789) and 98.73% with accession number: (MH777791). The lower nucleotide similarity was with *A. cornu* (87.95%; accession number: JF769451), *A. pipientis* (86.79%; accession numbers: MT943779) and *Australapatemon* spp. (83.9%; accession numbers: MH369768). In the phylogenetic trees (Figs. 6, 7) the *Bubulcus ibis* from this study clustered with *Renicola* spp. and *Apharyngostrigea* spp., as revealed by the analyses of COXI locus. The *Bubulcus ibis* digenean parasites strain formed a separated branch from the other *Renicolidae* spp. and *Strigeidae* spp.

### Pathological findings

**Figure 8:**
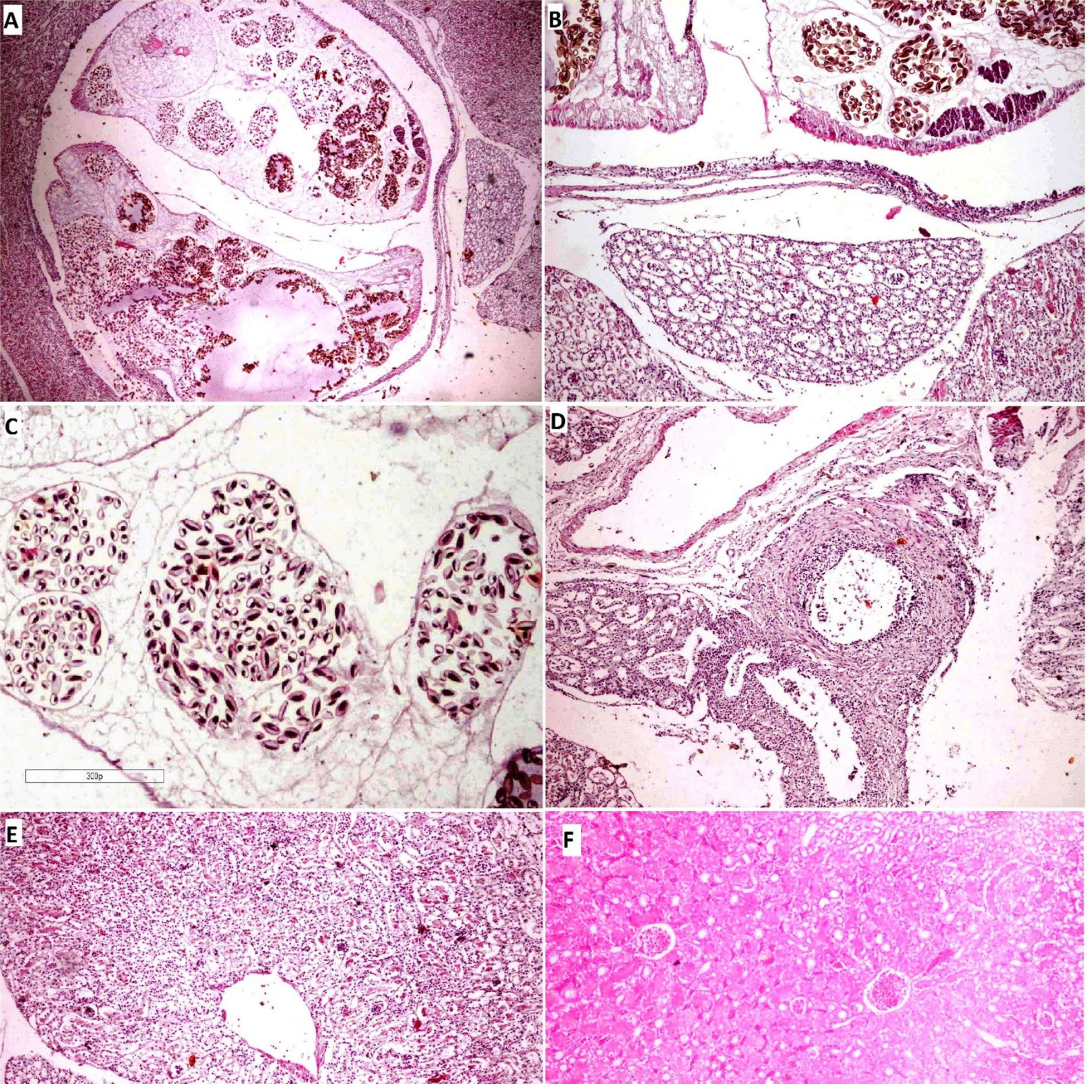
Macroscopical and histopathological picture of *Renicola* spp. infection in Cattle egret kidney. (**A**) Microphotograph of the heron kidney showing the presence of paired trematodes inside the parasitic cyst (H&E, 40x). (**B**) Microphotograph showing severe cystic dilatation, swelling and vacuolation of the renal tubule (H&E, 200x). (**C**) Kidney showing the presence of multiple numbers of eggs making pressure atrophy on renal tubules (H&E, 300x). (**D**) Microphotograph showing vasculitis of the renal blood vessel with thickening of its wall, perivascular edema and proliferation of fibroblasts. (**E**) Microphotograph showing diffuse degenerative changes of renal tubules and glomeruli with infiltration of inflammatory cells, note the cystic dilatation of the renal tubules in the center (H&E, 100x). (**F**) Microphotograph of the non-infected heron kidney showing the normal histological architecture of renal parenchyma (H&E, 200x).

Grossly the infected kidneys appeared enlarged and pale in color, with multiple dark nodules on their surface. These nodules contained two or more adult parasites and were surrounded by white yellowish caseated material and encapsulated by grey to the black thick well-defined capsule. Histopathological examination revealed the presence of considerable numbers of *Renicola* spp*.* (Fig. 8) in the renal parenchyma in the cortex and near the renal pelvis. The adult trematodes varied in size and contained great numbers of eggs of different sizes and shapes; they were ovoid with a basophilic stained curved body.

The parasites were surrounded by an inner cuboidal epithelial lining and outer delicate connective tissue fibers with few inflammatory cells, mainly macrophages and lymphocytes. The renal tubules near the parasitic nodules suffered from pressure atrophy. Some renal tubules showed granulation and vacuolation of their cytoplasm (Fig. 8), whereas others suffered from coagulative necrosis. Regenerating renal tubules were noticed in most cases. The intratubular stroma was densely infiltrated with inflammatory cells, mainly macrophages and lymphocytes. Free eggs of the parasites were seen in between the renal tubules. The renal blood vessels were congested with focal areas of hemorrhages near some blood vessels. Perivascular edema and aggregation of parasitic eggs were observed in most cases. Numerous eggs in the blood vessels suggest that they could be moved to other organs or stall in the blood vessels as emboli, causing significant problems in these birds.

## Discussion

The cattle egret is a valuable bird among cattle farmers due to its presumed role as a biocontrol of livestock parasites such as ticks and flies. In Egypt, the cattle egret (*Bubulcus ibis*) is an important bird and serves as a disease reservoir. Few researchers have discussed its significant contribution to disease transmission^28^. Renicolid trematodes are parasitizing tissues of kidneys and ureters of birds feeding on bivalves and fishes^29^. Worms although hermaphrodite normally live in pairs within dilated kidney tubules lined by cuboidal cells^13^. In this study, the total incidence of infected birds with *Renicola* spp. was 17.2%; this result disagreed with^30^ who recorded an infestation rate of 43.75% in Assiut. On the other side^13^, recorded an infestation rate of 12.4% in Iraq. The differences in the prevalence of *Renicola* spp. may be due to the availability of first (mainly crustaceans or mollusks) and second (fish) intermediate hosts.

The first trematode was of *Renicola* spp. morphologically resembles *R. goliath* but differ in the shape of the testes which appear as two large masses overlapping the ventral sucker while, in *Renicola* spp. it is lobed and lies side by side behind the ventral sucker. In the present specimens, the ovary appears highly branched and diagonally occupies a wide area with the testes. It appears as a deeply lobulated mass lying in front of the ventral sucker *Renicola*. The most outstanding feature that characterizes the first group of *Renicola* in comparison with *Renicola* spp. is its distribution of vitelline follicles. Vitellaria in the form of coarse follicles are in a linear distribution and occupy the two lateral fields of third third-fourth of the body, while, in the *Renicola* spp; it is distributed in the median and lateral fields of the intestinal caeca. Moreover, the other *Renicola* specimens morphologically resemble *R. ardeolae*^13^ but differ in the shape of the testes, it appears as a V-shaped form around the ventral sucker. This variation in the form of the testes was not described before. Where it appears as two branched testes and tandem in position; one anterior and one posterior to the ventral sucker in *R. ardeolae*^30^.

Vitellaria is in the form of coarse follicles in a pyramidal mass that occupy the lateral fields of the whole length of the intestinal caeca in *R. ardeolae*^30^. The present specimens, the vitelline follicles, are in a linear form and occupy the middle fourth of the two lateral fields. The specific name is derived from the locality and is considered a new species, of the third trematode species of *Apharyngostrigea* spp. differ from the homologous species, previously mentioned by^30^ where they described *A. ibis.*

In the present study, the morphological description of *Apharyngostrigea* spp. agreed with what was previously described by^3,20^ However, the present species differed from *Apharyngostrigea ibis*^31^ in the body length, length proportion between hind- and forebody and sizes of various organs, especially the proteolytic gland. Moreover^30^, described *A. magna* from *Ardea goliath*. Generally, in many helminths, the body surface plays an important role in integrating the worm's physiology with its micro-environments via nutrient assimilation, regulation of internal chemical pools by selective absorption or secretion mechanisms and chemosensory activity^32^. The results of the current investigation demonstrated that contact receptors covered the body surface of the *Apharyngostrigea*. Many different varieties of tegumental papillae may aid in feeding by expanding the absorption surface area. It also has a significant impact on the perception and selection of the materials to be ingested^3^. It is suggested that *Apharyngostrigea* external surface of the tegument might have other properties besides being absorptive; such as immunological protection and the maintenance of the worms in their habitat and assistance in retaining the position of the parasite within its host and mechanical stimulation of the host tissue for feeding purposes.

The present species is more related to *Apharyngostrigea ibis* (*A. ibis)* than *A. magna* where the major differences appear in the distribution of vitellaria in the hind body only in *A. ibis*, while it is present in the fore and hind body in the present specimens. The ovary takes the kidney in the present specimens but, it is rounded in *A. ibis*. Moreover, the distance between the tribocytic organ and ovary is large about 1.09 mm in the present specimens, while, it measures 0.21 mm. in *A. ibis* and ranges from 2.5 to 3.0 mm. in *A. magna.* The present specimens can be differentiated from *A. magna*^30^, in its three times smaller, Ratio of fore/hind body is 1.2 (1:3 in A magna) as well as the presence of suckers and characteristic proteolytic glands in the forebody of the specimens under discussion. Also, there is a difference in the locality of the herons^3^. These differences are enough to create the described as a new Spp. of *Apharyngostrigea*.

The phylogenetic analysis enabled us to identify the *Renicolidae* spp. and *Strigeidae* spp. found in this study. The sequence of COXI locus of *Renicola* spp. showed higher nucleotide similarity with^33^. The lower nucleotide similarity was with *R. buchanani*^34^, *R. lari*^7^ and *R. pinguis*^16^. *Apharyngostrigea* species were examined in this study as well as those with compatible COXI sequences in^17,18^. To confirm current and historical records of *Apharyngostrigea* spp. in South America, it would therefore be useful to get comparison sequences from Australia. Additional sequencing could also clarify historical records in North America^35^ and Africa^20^. According to^36^, there is little change in mitochondrial COXI between *Apharyngostrigea pipientis* and *A. cornu. A. cornu*’s potential existence in North America is supported analogously by the widespread distribution of *A. pipientis*^37^.

Our discussion was based on the statements of^12,15^, due to the limited availability of material on *Renicola* spp. and its impact on *Bubulcus ibis* tissues. In the current investigation, the kidneys displayed inflammatory cells in the intertubular stroma as well as degenerative alterations, including the necrosis of several renal tubules. These changes can be explained by the existence of circulating antigen toxins and immune complexes, which are potent initiators of inflammatory reactions because of parasitic infestation. The degenerative alterations and necrosis seen in this study are caused by circulating toxins^14^.

The endothelial damage caused by *Renicola* spp. eggs that were seen in the current study may have been directly caused by vasculitis, or they may have been caused indirectly by the impact of numerous toxic parasite products on the arteries wall. According to^38^, immune complexes that have accumulated in the vessel wall and caused damage may also be responsible for vasculitis. The hemorrhages and perivascular edema seen in this study can be explained by the involvement of the intima and endothelial lining of the blood arteries, which leads to vascular leakage and outflow of blood plasma and erythrocytes^15^.

## Conclusion

One of the most well-known herons in Egypt is the cattle egret. It is known as the farmer's friend since it benefits farmers and aids in their control of worms and insects. It serves as a disease reservoir. Therefore, Continuous monitoring programs are crucial tools for increasing our awareness of the abundance, variety, and ecological characteristics associated with several heron species. The findings of this study showed a higher incidence of *Renicola* spp. than *Apharyngostrigea* spp., as well as a strong correlation with renal impairment in Cattle egret. Through morphological and molecular biological analysis, the parasite was identified as *Renicola* spp. and *Apharyngostrigea* spp. Parasites are found in practically every population and interact with the host in a variety of ways. Further research analyzing the health of various heron species and environmental deterioration can help to close information gaps about the interactions between parasites, their hosts, and environmental health.

## Data Availability

All the authors declare that all the data supporting the results reported in our article were included in this article. The datasets generated or analyzed during the current study are available in the GENE BANK repository, accession numbers: [OR021986 and OQ955829].
